# Research on the impact of artificial intelligence technology on urban public health resilience

**DOI:** 10.3389/fpubh.2024.1506930

**Published:** 2025-01-07

**Authors:** Erdong Chen, Huaxin Zhang

**Affiliations:** School of Economics, Liaoning University, Shenyang, China

**Keywords:** artificial intelligence, invention patents, utility model patents, design patents, urban public health resilience, spatial effects

## Abstract

Urban public health resilience has become a critical focus in the transition to high-quality development, especially in addressing increasing public health challenges. This study explores the role of artificial intelligence (AI) technology in enhancing urban public health resilience across 284 Chinese cities from 2011 to 2021. Using a comprehensive index based on resistance, recovery, and innovation dimensions, the study quantifies AI technology levels through patent applications and authorizations, further disaggregating these into invention, utility model, and design patents. A two-way fixed effects regression model and spatial econometric models are employed to analyze the direct and spillover effects of AI on urban public health systems. The results demonstrate that AI technology significantly enhances resilience by improving resource allocation and response efficiency, with stronger impacts observed in eastern and central regions compared to western regions, where economic and technological capacities are weaker. Spatial analysis reveals significant positive spillover effects, particularly from patent authorizations, which enhance public health resilience in neighboring cities through cross-regional collaboration and resource sharing. Despite these advancements, regional disparities in economic development and technological infrastructure limit AI’s broader impact, underscoring the need for targeted policies, enhanced funding, and interdisciplinary training to bridge the digital divide. The findings highlight AI’s transformative potential in fostering urban public health resilience and call for sustained investment and collaboration to maximize its benefits.

## 1 Introduction

China is currently transitioning from rapid growth to high-quality development, and public health services, which are closely linked to the well-being of the population, have become a critical component of social welfare (1). The 20th National Congress of the Communist Party of China proposed the promotion of the Healthy China initiative, the improvement of the public health system, and the optimization of the public safety system. The Third Plenary Session of the 20th Central Committee emphasized the importance of promoting social co-governance, coordination between healthcare services and public health, and integration of medical care and prevention, bringing public health issues to the forefront of the national agenda. Meanwhile, as digital technology and the digital economy continue to develop, new-generation artificial intelligence (AI) is gradually permeating all aspects of social life. Particularly in the public health sector, AI has become an essential tool for enhancing the efficiency of public health governance (2, 3). Through big data analysis, machine learning, natural language processing, and predictive models, AI technology offers intelligent and data-driven solutions for public health, significantly improving the management efficiency, emergency response speed, and resource allocation of public health services (4, 5). However, regional disparities in public health resources have led to varying outcomes in the application of AI technology. Therefore, it is essential to rely on policy guidance and financial support to bridge the “digital divide” and address the uneven regional development of AI technology deployment, thereby enhancing urban public health resilience across all regions (6–8).

AI, with its capabilities surpassing human perception, reasoning, and learning, has become a driving force in economic, social, and technological development (8). Extensive academic discussions have explored the impact of AI across multiple fields, including regional economies, income distribution, employment, and industrial restructuring. While these studies provide theoretical and empirical analyses from different perspectives, their results have often been mixed (9–11). In the context of regional economic development, AI has demonstrated a positive impact by enhancing labor productivity and promoting economic growth through technological progress (12). However, AI may also contribute to structural unemployment and exacerbate income inequality (13). On the other hand, AI can reduce the demand for labor by automating certain tasks, while simultaneously creating new job opportunities, particularly in high-skill sectors, thereby increasing the demand for human capital (14). Additionally, AI promotes an innovation-driven development model, providing strong support for industrial transformation and upgrading, particularly in the digital economy and emerging industries. AI applications improve production efficiency, reshape production models in both traditional and emerging industries, and lead to substantial productivity gains (15). Research has shown that AI plays a significant role in expanding the breadth of innovation, cultivating new productivity, and promoting high-quality development (16). More importantly, AI offers new approaches in the public health field: it improves the workflows of medical equipment and healthcare personnel, enabling smart diagnostics and remote monitoring, thus reducing unnecessary patient-doctor interactions. Through big data collection, AI can more accurately predict disease trends and provide emergency plans for public health institutions. Additionally, AI can analyze vast amounts of patient data, learn from this data, and provide personalized medical recommendations for each patient, thereby meeting individual healthcare needs (17–19).

Resilience refers to a system’s ability to respond to external shocks and return to a stable state (20). In engineering, resilience describes a system’s capacity to recover to its original state after experiencing stress, emphasizing the balance achieved after responding to pressure (21). Ecological resilience posits that a system may enter a new, lower-level stable state after absorbing a certain level of disturbance, decline if unable to adapt to external shocks, or surpass its original state by restructuring to achieve a superior level (22). Drawing inspiration from adaptive system theory, scholars have revisited the concept of economic resilience, introducing the notion of “adaptive resilience” (23). Currently, there is a vast body of literature on economic resilience. Some scholars focus on measuring economic resilience using single indicators such as employment numbers, unemployment rates, wage levels, and GDP, while others adopt comprehensive indicator systems to evaluate regional economic resilience. Researchers have examined various factors influencing economic resilience, including how organizational culture and leadership affect resilience, and the role of industrial structure, the digital economy, and entrepreneurial activity in shaping economic resilience (24–26). Following major shocks such as the 2008 global financial crisis and the COVID-19 pandemic, countries and regions have conducted extensive research on how to enhance economic resilience and promote sustainable development, making this a key topic in regional development and macroeconomic policy research (27, 28).

A review of studies on AI technology and economic resilience reveals that AI has developed rapidly and expanded in scale, but still lags behind developed countries like the United States. In the field of urban public health resilience, research remains sparse, making it an area ripe for pioneering studies (29). This paper argues that existing research on AI technology and urban public health resilience is lacking, which has hindered the development of unified conclusions. First, there is a lack of clear definitions and explanations of the core concepts and connotations of urban public health resilience, making it difficult to draw consistent conclusions about AI’s impact and conduct systematic assessments. Second, there are discrepancies in the objective levels and practical scopes of the explanatory and dependent variables used to represent AI technology and urban public health resilience, affecting the robustness of regression results. Finally, the mechanism of interaction between the two has not yet been explored in either theoretical or empirical research. Based on this, the marginal contributions of this paper are as follows:

First, the study provides a comprehensive and systematic exploration of urban public health resilience, conceptualizing its core dimensions as resistance, recovery, and innovation (30, 31). It establishes an index system grounded in practical considerations, offering a robust theoretical framework and methodological foundation for advancing research in public health resilience. Second, this research employs patent application and authorization data related to artificial intelligence (AI) technologies as core explanatory variables. It further disaggregates patents into invention, utility model, and design categories, thereby quantifying the mechanisms through which AI influences urban public health resilience. This approach significantly enriches the quantitative methodologies in resilience studies (32). Third, to address existing gaps in the intersection of AI technology and public health resilience, the paper offers an innovative analysis of their underlying mechanisms. It demonstrates that AI technology substantially enhances urban public health resilience by improving healthcare efficiency, optimizing resource allocation, and fostering inter-city collaboration. Fourth, the study also conducts an in-depth investigation into the heterogeneous impacts of AI technology on public health resilience across eastern, central, and western Chinese cities. Additionally, it highlights the positive spatial spillover effects of AI technology, underscoring its capacity to benefit neighboring regions through knowledge transfer and resource sharing (33, 34).

The structure of this paper is as follows: Section 2 provides the theoretical analysis, Section 3 covers the sample and data, Section 4 discusses the results, Section 5 focuses on spatial tests, and the conclusion is presented in the final section.

## 2 Theoretical analysis

### 2.1 Public health resilience

Public health refers to the systematic and collective measures and strategies undertaken by nations and societies to improve population health. It involves various institutions, community groups, and individuals working in coordinated efforts and making scientific decisions to prevent diseases, extend human lifespan, and promote overall health (34). The main objectives of public health can be summarized into three areas: first, disease prevention through vaccination, health education, and improved healthcare infrastructure to mitigate potential health threats; second, early detection, timely treatment, and rehabilitation of existing diseases to minimize long-term health impacts; and third, the promotion of physical and mental well-being through daily habits and positive attitudes, enhancing overall quality of life, regardless of disease presence (35, 36).

Narrowly defined, public health resilience can be measured by the number of doctors, hospital beds, and healthcare workers. In a broader sense, it encompasses the multi-dimensional capacity of regional or urban public health systems to respond to external shocks—such as disease outbreaks, natural disasters, or environmental pollution—while focusing on core elements. This capacity includes three aspects: resistance, recovery, and innovation. Resistance refers to the ability of public health systems to reduce or withstand the negative impacts of external shocks through preventive measures, policy protections, and social support networks. Recovery is the system’s ability to swiftly restore core functions and re-establish normal operations after a crisis. Innovation reflects the system’s capacity to learn from crises, continuously improving and adapting to be more flexible and efficient when facing future challenges (37). Public health resilience is defined by its adaptability, resource diversity, systemic synergy, and capacity for early risk detection. By leveraging integrated social governance and optimized systems, it ensures the stability and sustainable advancement of urban public health infrastructures.

### 2.2 The role of AI technology in enhancing urban public health resilience

The self-evolving and integrative nature of AI technology has revolutionized knowledge production, strengthening connections between innovation elements and driving continuous AI iterations. This has blurred the boundaries between industries and has had a profound impact on urban public health services. As AI technology integrates further with traditional healthcare industries, its application in healthcare products and services has continued to grow, creating new value and significantly enhancing the operational efficiency and organizational effectiveness of healthcare institutions (38). Technological advancements, especially in AI and other emerging digital technologies, have had a far-reaching and positive impact on urban public health resilience through several pathways. Firstly, AI strengthens urban public health resilience. By employing data-driven analysis and sophisticated predictive models, AI can accurately forecast the transmission dynamics of infectious diseases. This capability provides early warning systems and robust risk prevention measures, enabling cities to respond more effectively to public health emergencies. Secondly, AI contributes to urban public health recovery. In the aftermath of a health crisis, AI-driven optimization of resources and intelligent allocation significantly improve recovery efficiency. It ensures the effective distribution of medical supplies and supports the development of personalized rehabilitation plans, streamlining patient recovery pathways. Moreover, public communication platforms powered by natural language processing reduce misunderstandings, helping populations adapt more smoothly to the post-crisis “new normal.” Finally, AI accelerates innovation in urban public health. By enabling seamless cross-sector collaboration, it facilitates the deep integration of medical and preventive systems. This integration drives the transformation of public health governance models toward intelligent, data-informed solutions, creating a foundation for smarter and more adaptive health management systems (39).

### 2.3 Regional heterogeneity of AI’s impact on urban public health resilience

Long-standing disparities exist among Chinese cities across various dimensions, and AI’s impact on urban public health resilience may exhibit regional heterogeneity. Understanding the level of AI development and its impact on urban public health resilience can assist policymakers in formulating specific policies to mitigate adverse shocks (40). Significant differences in economic development levels and technological innovation capabilities exist between the eastern, central, and western regions. In the eastern regions, with higher economic development, cities possess stronger technological innovation capabilities and greater capacity for public health resource allocation. As a result, AI applications can more effectively drive the optimization and management of public health systems, significantly enhancing urban public health resilience. Central cities, which are undergoing rapid industrialization and urbanization, have an urgent need for industrial transformation and upgrading. The introduction of AI technology can effectively elevate the technical capabilities of traditional healthcare industries, optimizing urban public health resource allocation and scheduling, with relatively stable technological gains. However, in the underdeveloped western regions, the weak economic foundation, insufficient technological innovation capacity, and scarce public health resources present significant challenges. Despite national policy support and resource input, the promotion and application of AI technology face numerous limitations, resulting in minimal impact on urban public health resilience (41).

### 2.4 Spatial correlation analysis of AI’s impact on urban public health resilience

The spatial spillover effects of AI technology on urban public health resilience are primarily reflected in cross-city technology diffusion, information sharing, and resource allocation. Firstly, spatial spillover theory underscores the importance of regional cooperation and coordinated management in enhancing urban public health resilience. Public health events such as disease outbreaks, environmental pollution, and emergency responses often transcend city boundaries, requiring collaborative efforts. While AI technology strengthens local public health systems, its positive effects spill over to neighboring cities, creating broader regional benefits (42). Secondly, information sharing and technological diffusion serve as key mechanisms for realizing these spatial effects. AI applications in healthcare, public health management, and environmental monitoring not only improve a city’s emergency response capabilities but also enable the transfer of technological advancements to neighboring cities. This is achieved through data sharing, cross-regional monitoring platforms, and the mobility of skilled professionals, fostering collective resilience across the region. Thirdly, the flow of production factors and collaborative effects provides essential support for technological spillovers. AI technology, which relies heavily on talent, capital, and infrastructure, facilitates the movement of these resources across regions. Early adopters of AI often attract high-skilled professionals and innovative enterprises, accelerating technology diffusion and optimizing resource allocation. This dynamic fosters the coordinated development of regional public health systems (43). Fourthly, improvements in a city’s public health resilience can generate a “demonstration effect” or “follow-up effect,” inspiring neighboring cities to adopt similar technologies and policies. These cascading influences encourage adjustments in regional policy frameworks and increased investments in technology, ultimately driving the collective optimization of public health systems across the region.

## 3 Sample and data

### 3.1 Sample selection

Prefecture-level cities in China serve as relatively comprehensive and autonomous administrative units. In 2011, a substantial accumulation of digital data related to public health was achieved, while significant breakthroughs were made in artificial intelligence technologies such as deep learning and big data analysis, marking the initial emergence of AI’s transformative potential. This study selects 284 prefecture-level cities and municipalities from 2011 to 2021 as the research sample, based on the availability and completeness of the data. The original data for this study primarily comes from the *China City Statistical Yearbook*, provincial and municipal statistical yearbooks, the *Urban Construction Statistical Yearbook*, prefecture-level city statistical bulletins, the China Research Data Service Platform (CNRDS), and the CEIC database. Where official data were unavailable, substitute indicators were applied, and missing data were imputed using linear interpolation.

### 3.2 Definition of variables

#### 3.2.1 Dependent variable

Urban public health resilience can be measured using either the single-index method or the comprehensive-index method. The single-index method is straightforward to calculate and easy to interpret; however, it often neglects the interactions and dynamic relationships within the system, which may introduce bias. In contrast, the comprehensive-index method provides a more holistic assessment by capturing the multidimensional nature of public health resilience and offering a systematic evaluation. Accordingly, this study employs the entropy weight method to construct a comprehensive index for measuring urban public health resilience. This study, grounded in scientific rigor, systematic analysis, and data availability, categorizes urban public health resilience into three dimensions: resistance, recovery, and innovation. To quantify these dimensions, specific secondary indicators were developed (44). Given the differences in scale and orientation among these indicators, the range standardization method was employed to mitigate their impact on the results. Subsequently, weights were assigned using the coefficient of variation method, resulting in a comprehensive index of urban public health resilience. Table 1 outlines the secondary indicators, along with their corresponding descriptions and attributes, used in the comprehensive index methodology.

**Table 1:** Comprehensive evaluation index system for urban public health resilience.

Primary dimension	Secondary indicator	Indicator definition	Attribute
Resistance	Basic Pension Insurance Coverage Rate	Ratio of people covered by pension insurance to the resident population	+
	Basic Medical Insurance Coverage Rate	Ratio of people covered by medical insurance to the resident population	+
	Unemployment Insurance Coverage Rate	Ratio of people covered by unemployment insurance to the resident population	+
Recovery	Number of Hospitals	Number of hospitals per 10,000 people	+
	Number of Beds	Number of beds per 100 people	+
	Number of Doctors	Number of doctors per 100 people	+
Innovation	Education Expenditure	Ratio of education expenditure to GDP	+
	R&D Expenditure	Ratio of R&D expenditure to DDP	+
	Innovation	Number of newly established enterprises per 100 people	+

#### 3.2.2 Independent variable

AI technology reflects a city’s capacity for technological innovation and knowledge accumulation. Innovations in AI algorithms, applications, and system architectures are often represented and safeguarded through patents (44). Artificial intelligence patents represent a tangible indicator of technological innovation, offering a robust and comprehensive data foundation for empirical research. They serve as a reliable proxy for evaluating the advancements and maturity of artificial intelligence technologies, aligning with rigorous academic standards. In this study, AI patents are used as a proxy variable to measure AI technology levels. Using the IPC classification, data on AI patent applications and authorizations were collected from the Chinese patent database via Python web-scraping techniques, with separate regressions conducted for each, followed by interaction tests. Additionally, AI patents were classified to further explore the mechanisms and impacts of AI technology on urban public health resilience.

#### 3.2.3 Control variables

Government intervention (gov) is measured by the ratio of local fiscal general budget revenue to GDP, with higher values indicating stronger government intervention in the market. Openness to trade (tra) is measured by the ratio of imports and exports, converted to RMB using the prevailing exchange rate, to GDP. Excessive reliance on trade may increase a city’s vulnerability to external public health shocks. Population density is measured by the logarithm of the urban resident population. When effectively planned and managed, higher population density can significantly enhance a city’s response capacity for public health resilience and improve the efficiency of human capital allocation. Resident income is measured by the ratio of disposable income to the resident population. Higher income levels can strengthen a city’s ability to handle sudden public health crises and improve long-term resilience. Electricity consumption (elec) is measured by the ratio of total electricity consumption to the resident population. A stable power supply improves residents’ quality of life and public health resilience. Energy efficiency (eng) is measured by the ratio of standard coal consumption to GDP. Although improvements in energy efficiency may yield short-term benefits, they can have a long-term negative impact on urban public health resilience. The definitions of these variables are provided in Table 2.

**Table 2:** Main variable definition.

Variable type	Variable name	Abbreviation	Definition
Dependent variable	Urban public health resilience	res	Entropy-weighted composite index
Independent variables	AI patent applications	lnaiat	Logarithm of the number of AI patent applications
	AI invention patent applications	lnaiaf	Logarithm of the number of AI invention patent applications
	AI utility model patent applications	lnaias	Logarithm of the number of AI utility model patent applications
	AI design patent applications	lnaiaw	Logarithm of the number of AI design patent applications
	AI patents granted	lnaigt	Logarithm of the number of AI patents granted
	AI invention patents granted	lnaigf	Logarithm of the number of AI invention patents granted
	AI utility model patents granted	lnaigs	Logarithm of the number of AI utility model patents granted
	AI design patents granted	lnaigw	Logarithm of the number of AI design patents granted
Control variables	Government intervention	gov	Budgetary revenue to GDP
	Openness to trade	tra	Total imports and exports to GDP
	Population density	pop	Logarithm of the resident population
	Residents income	town	Per capita disposable income of residents to Resident population
	Electricity consumption	elec	Total electricity consumption to Resident population
	Energy efficiency	eng	Standard coal consumption to GDP

### 3.3 Model construction

This study constructs the following basic regression model:


(1) $$res_{\text{it}}=a_{0}+a_{1}AI_{\text{it}}+\sum_{j}a_{j}\times X+u_{t}+\lambda_{i}+\varepsilon_{\text{it}}$$

where *res* represents urban public health resilience, *AI* refers to artificial intelligence technology, which, during the analysis, can be substituted by the natural logarithm of patent applications, authorizations, or categorized patents. The subscript *i* denotes the city, and *t* denotes the year. *X* represents the control variables, $u_{t}$ accounts for time-fixed effects, $\lambda_{i}$ for individual-fixed effects, $\varepsilon_{\text{it}}$ is the random disturbance term, and *α* denotes the regression coefficients.

To explore the spatial effects of AI technology on urban public health resilience, the study employs a Spatial Autoregressive Model (SAR) Equation 2 and a Spatial Error Model (SEM) Equation 3:

Spatial Autoregressive Model (SAR):


(2) $$res_{\text{it}}=\rho W\cdot res_{\text{it}}+\beta X_{\text{it}}+\mu_{t}+\lambda_{i}+\varepsilon_{\text{it}}$$

where *ρ* is the spatial autoregressive coefficient, *W* is the spatial weight matrix, β represents the corresponding influence coefficient, and the other variables retain the same meanings as in Equation 1.

Spatial Error Model (SEM):


(3) $$res_{\text{it}}=\gamma X_{\text{it}}+\mu_{t}+\lambda_{i}+\varepsilon_{\text{it}}\;\varepsilon_{\text{it}}=\delta W\cdot \varepsilon_{\text{it}}+\nu_{\text{it}}$$

where δ is the spatial error coefficient, W is the spatial weight matrix, $\varepsilon_{\text{it}}$ represents the impact of error shocks from neighboring regions’ public health resilience on the current region, $\nu_{\text{it}}$ is the random error term, γ denotes the regression coefficients, and the other variables are defined as in Equation 1.

The statistical descriptions of the variables are presented in Table 3. The standard deviation of urban public health resilience is close to the mean, and there is a notable difference between the minimum and maximum values, indicating variability in the levels of public health resilience across the sample cities. For the AI technology variables—including patent applications, invention patent applications, utility model patent applications, design patent applications, as well as patent authorizations, invention patent authorizations, utility model patent authorizations, and design patent authorizations—the standard deviations are smaller than the mean, with a noticeable difference between the minimum and maximum values. This suggests a degree of variation in AI technology across cities, aligning with the actual progress of AI development. Population density, resident income, and electricity consumption are at relatively high levels, while openness to trade and energy efficiency are at moderate levels. Government intervention is at a relatively low level, providing empirical support for the use of a two-way fixed effects model.

**Table 3:** Descriptive statistical analysis.

Variable	Obs	Mean	Std. Dev.	Min	Max
res	3,124	0.038	0.044	0.009	0.656
lnaiat	3,124	7.530	1.296	2.773	12.476
lnaiaf	3,124	6.860	1.300	2.079	11.789
lnaias	3,124	6.313	1.306	1.386	11.352
lnaiaw	3,124	5.855	1.302	1.386	10.712
lnaigt	3,124	5.856	1.318	1.386	11.390
lnaigf	3,124	5.230	1.318	0.693	10.710
lnaigs	3,124	4.584	1.323	0.000	10.142
lnaigw	3,124	4.139	1.336	0.000	9.812
gov	3,124	0.077	0.027	0.023	0.227
tra	3,124	0.176	0.283	0.000	2.491
pop	3,124	5.725	0.990	1.736	9.420
town	3,124	2.338	0.476	1.207	4.626
elec	3,124	4.043	1.134	0.105	7.467
eng	3,124	0.136	0.150	0.008	2.283

## 4 Estimation and result

### 4.1 Benchmark regression analysis

Before empirically examining the relationship between AI technology and urban public health resilience, it is essential to assess the multicollinearity and stationarity of the variables to avoid spurious regression. The variance inflation factor (VIF) results indicate an average VIF of 1.52, with a maximum value of 1.92, well below the threshold of 10, suggesting no multicollinearity among the variables. The Hausman test results reject the null hypothesis at the 1% significance level, confirming that the two-way fixed effects model is appropriate for the benchmark regression analysis.

This study uses panel data from 284 cities between 2011 and 2021 to conduct benchmark regression analysis with a two-way fixed effects model, as presented in Table 4. Columns (1) and (5) display the results of univariate regressions, where the regression coefficients are positive and significant at the 1% level, indicating that the number of AI patent applications and authorizations positively influences urban public health resilience. Studies have demonstrated that artificial intelligence (AI) technology significantly enhances urban public health resilience by leveraging data-driven methodologies, intelligent optimization, and advanced monitoring systems. These capabilities collectively improve the responsiveness, precision, and sustainability of public health infrastructures, enabling more effective management of emerging challenges in urban health ecosystems. Columns (2)–(4) and (6)–(8) apply stepwise regression, incorporating control variables such as government intervention, openness to trade, population density, resident income, electricity consumption, and energy efficiency. Table 4 shows that the regression coefficients remain positive and significant at the 1% level across these models. The findings demonstrate a significant and stable positive relationship between AI patent applications, authorizations, and urban public health resilience, which aligns with theoretical expectations. The study reveals that AI technology contributes to strengthening the public health resilience of urban entities by enhancing their ability to adapt to external environmental changes. In times of shock, cities can continuously adjust their operational models, utilizing AI to perceive changes in the external environment and make adaptive modifications, thereby enhancing resilience, flexibility, and gaining new developmental advantages, leading to quicker and more effective recovery from challenges (45).

**Table 4:** Basic regression results.

Variables	res							
	(1)	(2)	(3)	(4)	(5)	(6)	(7)	(8)
lnaiat	0.007***	0.007***	0.008***	0.007***				
	(0.003)	(0.003)	(0.003)	(0.003)				
lnaigt					0.014***	0.015***	0.016***	0.014***
					(0.004)	(0.004)	(0.004)	(0.004)
gov		0.248***	0.265***	0.280***		0.264***	0.282***	0.295***
		(0.058)	(0.057)	(0.057)		(0.058)	(0.057)	(0.057)
tra		−0.025***	−0.021***	−0.017**		−0.027***	−0.022***	−0.018**
			(0.008)	(0.008)		(0.008)	(0.008)	(0.008)
pop			0.031***	0.029***			0.032***	0.030***
			(0.010)	(0.010)			(0.010)	(0.010)
town			0.031***	0.031***			0.031***	0.031***
			(0.005)	(0.005)			(0.005)	(0.005)
elec				0.008***				0.007**
				(0.003)				(0.003)
eng				−0.041***				−0.040***
				(0.008)				(0.008)
Constant	−0.001	−0.012	−0.283***	−0.289***	−0.020	−0.041**	−0.313***	−0.314***
	(0.018)	(0.018)	(0.060)	(0.060)	(0.019)	(0.020)	(0.060)	(0.061)
Observations	3,124	3,124	3,124	3,124	3,124	3,124	3,124	3,124
R-squared	0.392	0.494	0.667	0.753	0.408	0.513	0.695	0.776
Control variables	YES	YES	YES	YES	YES	YES	YES	YES
Individual effect	YES	YES	YES	YES	YES	YES	YES	YES
Time effect	YES	YES	YES	YES	YES	YES	YES	YES

Standard errors in parentheses (***p < 0.01, **p < 0.05, *p < 0.1).

Regarding the control variables, the coefficients for government intervention, population density, resident income, and electricity consumption are significantly positive, indicating their beneficial impact. High levels of government intervention can stabilize economic fluctuations through fiscal policies, thereby strengthening the stability and resilience of urban public health services. In cities with higher population density, healthcare resources tend to be more concentrated, creating agglomeration effects that enable faster and more convenient access to healthcare services, thus enhancing urban public health resilience. Higher resident income levels contribute to raising public health awareness and participation, further strengthening urban public health resilience. A reliable electricity supply improves cities’ ability to respond to public health emergencies, ensuring that public health activities can quickly return to normal. In contrast, the coefficients for openness to trade and energy efficiency are significantly negative, indicating an inhibitory effect. While openness to trade can drive economic growth, it also increases the risk of infectious disease transmission from external sources, creating more complex disease control challenges and weakening the adaptability of urban public health services. Excessive emphasis on energy efficiency can divert funds and resources from public health investments, leading to imbalanced resource allocation and reducing the emergency response capacity of public health services during crises.

### 4.2 Lagged effect analysis

As a new-generation digital economy innovation, AI technology has developed rapidly but faces delays in widespread practical application. This lag is attributed to several factors, including the inability of algorithmic models to fully keep pace with evolving business needs, the time required to accumulate complementary technological investments, and the lack of fully mature policies, regulations, and ethical frameworks necessary for widespread AI adoption across various sectors. To account for these factors, this study uses the one-period and two-period lags of the AI technology variable as explanatory variables in the regression analysis, with the results presented in Table 5. Columns (1)–(4) in Table 5 show that the one-period and two-period lagged regression results for AI patent applications and authorizations remain significantly positive. While the correlation coefficients decrease slightly, the results remain robust, indicating that AI technology continues to exert a sustained positive influence on the development of urban public health resilience. The influence of artificial intelligence (AI) technology variables is observed to diminish gradually over longer lag periods. This phenomenon can be attributed to the initially pronounced impact of AI technologies, which tend to exhibit significant effectiveness during the early stages of adoption. However, as time progresses, their marginal benefits decrease, with subsequent effects increasingly dependent on the interplay of other evolving factors.

**Table 5:** Regression results of lagged variables on urban public health resilience.

	(1)	(2)	(3)	(4)
Variables	res	res	res	res
L.lnaiat	0.007***			
	(0.003)			
L2.lnaiat		0.006**		
		(0.003)		
L.lnaigt			0.010**	
			(0.004)	
L2.lnaigt				0.008**
				(0.004)
Constant	−0.441***	−0.413***	−0.448***	−0.414***
	(0.071)	(0.073)	(0.071)	(0.073)
Observations	2,839	2,555	2,839	2,555
R-squared	0.638	0.657	0.638	0.657
Control variables	YES	YES	YES	YES
Individual Effect	YES	YES	YES	YES
Time Effect	YES	YES	YES	YES

Standard errors in parentheses (***p < 0.01, **p < 0.05, *p < 0.1).

### 4.3 Endogeneity treatment: instrumental variable method

There may be an endogeneity issue in the relationship between AI technology and urban public health resilience. To address this, the study uses the average AI technology variable of different cities within the same province in the same year as an instrumental variable. The average level of artificial intelligence (AI) technology across cities within the same province reflects regional trends in AI development and is strongly correlated with the intensity of local economic activities. This alignment satisfies the relevance criterion for instrumental variables. Moreover, the average AI technology level in other cities within the province does not directly influence the dependent variable of the target city. Instead, its impact is mediated through its influence on the AI technology level of the target city, thereby meeting the exogeneity requirement for instrumental variables. Table 6 presents the results of the two-stage least squares (2SLS) regression. For the identification of the instrumental variable, the Anderson canon. Corr. LM statistic for AI patent applications and authorizations significantly rejects the null hypothesis of “under-identification of the instrumental variable” at the 1% level. The Cragg-Donald Wald F statistic for AI patent applications and authorizations passes the weak instrumental variable test, confirming that the chosen instrumental variable is valid. According to the second-stage regression results in Table 6, the coefficient for AI patent applications is 0.008 and significant at the 1% level, while the coefficient for AI patent authorizations is 0.015 and also significant at the 1% level. These findings indicate that AI technology positively contributes to urban public health resilience, confirming the robustness of the study’s conclusions.

**Table 6:** Instrumental variable: 2SLS regression results.

Variables	IV1		IV2	
	First stage	Second stage	First stage	Second stage
lnaiativ	0.996***			
	(0.003)			
lnaiat		0.008***		
		(0.003)		
lnaigtiv				
			
lnaigt			0.092***	0.015***
			(0.005)	(0.004)
F		77638.43		38392.04
Anderson canon. Corr. LM statistic		2692.854		1331.119
Cragg-Donald Wald F statistic		0		0
Observations	3,068	3,068	3,068	3,068
R-squared	0.992	0.782	0.993	0.793
Control variables	YES	YES	YES	YES
Individual Effect	YES	YES	YES	YES
Time Effect	YES	YES	YES	YES

Standard errors in parentheses (***p < 0.01, **p < 0.05, *p < 0.1).

### 4.4 Robustness tests

#### 4.4.1 Static panel regression

##### 4.4.1.1 Alternative estimation method

The previously calculated values of urban public health resilience are non-negative and exhibit a truncated distribution with a lower bound at zero. To address this, a two-way fixed-effects panel Tobit model was employed for re-estimation. As shown in column (1) of Table 7, the regression coefficient for the AI technology variable remains significantly positive at the 1% level, confirming the robustness of the baseline regression.

**Table 7:** Robustness tests of static panel regression.

Variables	(1)		(2)		(3)	
	Change the estimation method		Exclude the municipality sample		Winsorization test	
lnaiat	0.016***		0.007***		0.006***	
	(0.004)		(0.003)		(0.002)	
lnaigt		0.011***		0.014***		0.012***
		(0.003)		(0.004)		(0.002)
Constant	−0.002***	−0.026***	−0.354***	−0.399***	−0.324***	−0.347***
	(0.005)	(0.004)	(0.071)	(0.072)	(0.040)	(0.041)
Observations	3,124	3,124	3,079	3,079	3,124	3,124
R-squared	/	/	0.775	0.792	0.781	0.786
Control variables	YES	YES	YES	YES	YES	YES
Individual Effect	YES	YES	YES	YES	YES	YES
Time Effect	YES	YES	YES	YES	YES	YES

Standard errors in parentheses (***p < 0.01, **p < 0.05, *p < 0.1).

##### 4.4.1.2 Excluding municipality samples

Given the substantial differences in economic scale between municipalities and regular prefecture-level cities, as well as the discrepancies in AI technology levels and urban public health resilience, excluding municipality samples minimizes the distortion caused by these economic differences and yields more targeted analysis. The results in column (2) of Table 7 indicate that after excluding the municipalities, the regression remains significant, with the positive influence of the AI technology variable on urban public health resilience unchanged. The regression coefficients show slight adjustments, suggesting that the inclusion of municipalities had some effect on the overall results.

##### 4.4.1.3 Winsorization test

The Winsorization test aims to replace extreme values in the data with more reasonable upper and lower bounds to reduce the impact of outliers on the model results. Column (3) of Table 7 presents the regression results after Winsorization. The results remain significant, with minor changes to the coefficients compared to the original regression, indicating that the influence of extreme values on the original results has been mitigated after Winsorization.

#### 4.4.2 Dynamic panel regression

To further verify the impact of AI technology on urban public health resilience, this study incorporates a lagged term of urban public health resilience to construct a dynamic panel, using the SYS-GMM method for regression. The results are presented in Table 8. The regression coefficient for the AI technology variable remains significantly positive, indicating a substantial positive effect of AI technology on urban public health resilience. The AR (1) autocorrelation is significant, while AR (2) autocorrelation is not, confirming the absence of second-order autocorrelation issues in the model. Furthermore, the results of the Sargan test indicate that the overall estimation possesses strong explanatory power and robustness, satisfying the requirements for causal statistical inference and aligning with theoretical expectations.

**Table 8:** Robustness test of dynamic panel: SYS-GMM regression.

Variables	res	
	(1)	(2)
L.res	0.239***	0.232***
	(0.078)	(0.083)
lnaiat	0.007*	
	(0.004)	
lnaigt		0.018** (0.009)
Observations	2,833	2,833
Control variables	YES	YES
Number of Instrumental Variables	49	49
AR (1)	−3.07	−3.18
AR (2)	0.27	−0.11
Sargan test of overid	2772.85	4.22

Standard errors in parentheses (***p < 0.01, **p < 0.05, *p < 0.1).

### 4.5 Heterogeneity analysis

#### 4.5.1 Heterogeneity in the impact of AI technology on urban public health resilience

Based on the economic regional divisions provided by the National Bureau of Statistics, this study categorizes the 284 cities into eastern, central, and western regions, conducting group regressions according to model (1). The regression results for the AI technology variable are presented in Table 9. The heterogeneity analysis reveals significant differences in the impact of AI technology on urban public health resilience across China’s economic regions. In the eastern region, both AI patent applications and authorizations have a significantly positive effect on urban public health resilience. The central region demonstrates the most stable technological benefits, with both the current and lagged effects of patent applications and authorizations being significant. In contrast, no significant relationship is observed between AI patent applications, authorizations, and urban public health resilience in the western region.

**Table 9:** Heterogeneity analysis of AI technology variables.

		(1)	(2)	(3)	(4)	(5)	(6)
	Variables	res	res	res	res	res	res
		Eastern region		Central region		Western region	
Number of AI patent applications	lnaiat	0.014***		0.014***		−0.006	
		(0.005)		(0.003)		(0.006)	
	L.lnaiat		0.006		0.013***		0.004
			(0.005)		(0.003)		(0.006)
	Constant	−0.278***	−0.639***	−0.185***	−0.191***	−0.213	−0.416**
		(0.090)	(0.129)	(0.070)	(0.062)	(0.186)	(0.194)
	Observations	1,099	999	1,100	1,000	924	840
	R-squared	0.636	0.784	0.636	0.784	0.796	0.784
Number of AI patent authorizations	lnaigt	0.020**		0.019***		−0.008	
		(0.008)		(0.004)		(0.009)	
	L.lnaigt		0.011		0.012***		−0.003
			(0.008)		(0.004)		(0.009)
	Constant	−0.296***	−0.679***	−0.206***	−0.196***	−0.219	−0.373**
		(0.095)	(0.134)	(0.070)	(0.063)	(0.185)	(0.190)
	Observations	1,099	999	1,100	1,000	924	840
	R-squared	0.587	0.678	0.742	0.689	0.786	0.875

Standard errors in parentheses (***p < 0.01, **p < 0.05, *p < 0.1).

#### 4.5.2 Heterogeneity in the impact of AI technology categories’ patent authorizations on urban public health resilience

The regional heterogeneity of AI technology arises from differences in the dominant technology categories across regions and their respective mechanisms of influence. The distribution of AI technology patent authorizations by category from 2011 to 2021 is presented in Table 10. In the eastern region, due to factors such as the concentration of innovation resources, industrial structure, talent advantages, internationalization, and industrial cluster effects, patent authorizations across all three major categories hold a distinct advantage, significantly surpassing those in the central and western regions. The patent structures across regions are notably similar, with invention patents dominating in all areas, followed by utility model patents, and design patents representing the smallest proportion. The eastern region stands out with a substantially higher number of patents, particularly in invention patent authorizations, compared to the central and western regions.

**Table 10:** Regional distribution of patent authorizations by AI technology categories.

	Invention patent		Utility model patent		Design patent	
	Number of authorizations	Percentage (%)	Number of authorizations	Percentage (%)	Number of authorizations	Percentage (%)
Eastern region	1,194,785	53.54	620,794	27.82	415,628	18.62
Central region	228,948	53.51	123,336	28.82	75,534	17.65
Western region	158,426	53.12	84,411	28.30	55,384	18.57

To further understand the drivers and trends of AI technology development, this study conducts a detailed analysis using the number of patent authorizations across various AI technology categories, as shown in Table 11.

**Table 11:** Heterogeneity analysis of AI invention patents, utility model patents, and design patents authorizations.

	Eastern region		Central region				Western region
	(1)	(2)	(3)	(4)	(5)	(6)	(7)
	Current period	First lag	Current period	First lag	Second lag	Third lag	Current period
lnaigf	0.016**	0.007	0.016***	0.010***	0.008***	0.005	−0.007
	(0.007)	(0.007)	(0.003)	(0.003)	(0.003)	(0.005)	(0.007)
lnaigs	0.005	/	0.012***	0.008***	0.011***	0.005	−0.007
	(0.006)	/	(0.003)	(0.003)	(0.003)	(0.003)	(0.007)
lnaigw	0.014**	0.003	0.012***	0.004	/	/	−0.007
	(0.005)	(0.005)	(0.003)	(0.003)	/	/	(0.007)
Observations	1,100	1,000	1,100	1,000	900	800	924
Control variables	YES	YES	YES	YES	YES	YES	YES
Individual Effect	YES	YES	YES	YES	YES	YES	YES
Time Effect	YES	YES	YES	YES	YES	YES	YES

Standard errors in parentheses (***p < 0.01, **p < 0.05, *p < 0.1).

In the eastern region, the number of AI invention patent authorizations has a direct and positive impact on urban public health resilience in the current period. This may be due to the eastern region’s position as a core area for technological innovation and economic development, benefiting from well-established research infrastructure and industrial chains. In the central region, invention patent authorizations positively influence urban public health resilience both in the current period and in the first and second lagged periods. This can be attributed to the central region’s focus on patent technology layout, market demand, and application, coupled with relatively well-developed institutions, enabling the rapid realization of the economic value of invention patents. In contrast, in the western region, the benefits of AI invention patents remain minimal, resulting in an insignificant impact on urban public health resilience. This underscores the limited contribution of such patents to enhancing public health resilience at this stage. The likely reasons include insufficient innovation investment, constrained by weak economic foundations, underdeveloped technological infrastructure, and suboptimal policy implementation. These factors collectively impede the effective application of AI technologies and the advancement of management practices.

For AI utility model patent authorizations, no significant correlation is found with urban public health resilience in the eastern region. In the central region, utility model patent authorizations positively impact urban public health resilience in the current period, as well as in the first and second lagged periods. In the western region, no correlation is observed between the two.

AI design patent authorizations positively affect urban public health resilience in the current period in the eastern region. Similarly, a positive effect is observed in the central region during the same period. However, no significant correlation is found between AI design patent authorizations and urban public health resilience in the western region.

## 5 Further analysis: spatial effects of AI technology on urban public health resilience

AI technology not only strengthens urban public health resilience by improving coordination and collaboration in public health activities within a region but also promotes cross-regional development through the free movement of production factors such as capital, labor, and technology. To verify this hypothesis, this study employs spatial econometric models to analyze the spatial effects of local AI technology development on the public health resilience of neighboring cities. The aim is to determine whether technological advancements can transcend geographic and economic boundaries, producing positive impacts on nearby cities.

### 5.1 Spatial autocorrelation test

In this study, a spatial inverse distance weight matrix is constructed. The spatial inverse distance weight matrix is a tool used in spatial econometrics to represent geographic relationships between regions, typically applied to describe spatial interdependencies among regions or cities. The core principle is that regions closer to one another exert a greater influence, while regions farther apart have a smaller impact. The weights are based on the geographic distance between regions, with weights inversely proportional to the distance. This method is widely used in spatial autoregressive models (SAR) and spatial error models (SEM) to capture the mutual influence and spatial dependence between regions. The calculation Equation 4 is as follows:


(4) $$\omega_{ij}=1/\delta_{ij},\text{ for }i\neq j$$

where $\delta_{ij}$ represents the distance between region i and region j, and $\omega_{ij}$ represents the spatial weight of region i on region j. The smaller the distance, the greater the weight. When i = j(i.e., within the same region), the spatial weight is set to zero or omitted to ignore self-influence. To ensure suitability for spatial regression analysis, the weight matrix is typically normalized so that the sum of weights for each region equals 1, representing the relative strength of influence each region has on others.

Prior to spatial analysis, it is crucial to rigorously test for spatial autocorrelation to reveal spatial dependencies that may be overlooked in traditional econometric models, thus preventing biased estimation results. Moran’s I is frequently used for this purpose, as it effectively reflects the degree of spatial correlation. This study employs the Moran’s I index for the analysis. Table 12 presents the Moran’s I index for urban public health resilience from 2011 to 2021, using the spatial inverse distance weight matrix. The results indicate that the Moran’s I index is consistently greater than 0 and statistically significant, suggesting a strong positive spatial autocorrelation in urban public health resilience. This underscores the necessity of applying spatial econometric models.

**Table 12:** Moran’s I index.

Year	2011	2012	2013	2014	2015	2016	2017	2018	2019	2020	2021
Moran’s I	0.014	0.019	0.018	0.020	0.014	0.019	0.076	0.083	0.021	0.094	0.046
	(0.000)	(0.000)	(0.000)	(0.000)	(0.000)	(0.000)	(0.000)	(0.000)	(0.000)	(0.000)	(0.000)

### 5.2 Spatial impact of AI technology on urban public health resilience

The spatial autocorrelation analysis confirms the presence of significant spatial dependence in urban public health resilience. To further explore this, spatial econometric models (2) and (3) were constructed for analysis. The regression results presented in Table 13 indicate the following insights regarding AI patent applications and grants. For the number of AI patent applications, the Log-Likelihood statistic for the Spatial Autoregressive Model (SAR) is 6060.758, compared to 6036.019 for the Spatial Error Model (SEM), leading to the selection of the SAR model. The goodness of fit for the SAR model is 0.823, whereas for the SEM model, it is 0.752, further reinforcing the preference for the SAR model. For the number of AI patent grants, the Log-Likelihood statistic for the SAR model is 6040.824, slightly higher than 6037.139 for the SEM model, once again supporting the selection of the SAR model. Additionally, the goodness of fit for the SAR model is 0.657, compared to 0.568 for the SEM model, confirming the SAR model as the better choice. Based on these results, this study employs the fixed-effects Spatial Autoregressive Model to analyze the impact of AI technology on urban public health resilience (46).

**Table 13:** Spatial model regression results.

	(1)		(2)	(3)
	SAR model		SEM model	
Variables	lnaiat	lnaigt	lnaiat	lnaigt
Patent variables	0.006**	0.011***	0.006**	0.011***
	(0.002)	(0.004)	(0.003)	(0.004)
rho	0.530***	0.496***		
	(0.103)	(0.108)		
lambda			0.475***	0.420***
			(0.113)	(0.124)
sigma2_e	0.001***	0.001***	0.001***	0.001***
	(0.000)	(0.000)	(0.000)	(0.000)
Log-likelihood	6060.758	6040.824	6036.019	6037.139
Direct	0.006**	0.011***		
	(0.002)	(0.004)		
Indirect	0.007	0.011*		
	(0.004)	(0.006)		
Total	0.013**	0.022***		
	(0.006)	(0.009)		
Observations	3,124	3,124	3,124	3,124
R-squared	0.823	0.657	0.752	0.568

Standard errors in parentheses (***p < 0.01, **p < 0.05, *p < 0.1).

Following the setup of model (2), the regression results are shown in Table 13. Under the spatial inverse distance weight matrix, the coefficient for AI patent applications is 0.006 and significantly positive at the 5% level, while the coefficient for AI patent authorizations is 0.011 and significantly positive at the 1% level. These findings align with the baseline regression, indicating that AI technology fosters the development of urban public health resilience. The spatial autoregressive coefficients (rho) for both AI patent applications and authorizations are significantly positive at the 1% level, suggesting a positive spatial spillover effect. The study finds that public health events, such as epidemics or environmental health issues, often have a cross-regional nature, requiring inter-regional cooperation in public health management and response. AI technology facilitates real-time information sharing, data analysis, and emergency response across cities, thereby enhancing public health resilience throughout the region.

To further examine the spatial effects of AI technology on urban public health resilience, this study decomposes the spatial effects. Table 13 reports the direct, indirect, and total effects of AI technology on urban public health resilience under the spatial inverse distance weight matrix. The direct effect reflects the impact of AI technology on public health resilience within the local region, while the indirect effect captures its influence on neighboring regions. The regression results show that for the direct effect, AI patent applications are significantly positive at the 5% level, and AI patent authorizations are significantly positive at the 1% level, indicating that AI technology positively contributes to public health resilience in the local city. This may be because AI technology can analyze medical records, social media data, and environmental sensor data to predict disease transmission paths and affected areas, enabling public health institutions to respond more swiftly. Regarding the indirect effect, AI patent applications exhibit a positive but not significant impact, whereas AI patent authorizations are significantly positive at the 10% level. This suggests that AI patent authorizations have a significant positive impact on the public health resilience of neighboring cities, while the influence of AI patent applications, though positive, is not statistically significant. This could be because public health resilience depends on the reliability and stability of the technology. Unapproved patent technologies, lacking practical validation, may not function consistently in critical public health scenarios, limiting their spatial spillover effects. Conversely, authorized patents, having undergone rigorous review and validation, are more likely to provide stable and reliable public health support, helping cities respond effectively to emergencies such as pandemics or natural disasters, thereby demonstrating clear spatial spillover effects. For the total effect, both AI patent applications and authorizations are significantly positive at the 5 and 1% levels, respectively. This indicates that AI technology has an overall positive effect on urban public health resilience, reflecting the combined influence of direct and indirect effects. Research demonstrates that the adoption of artificial intelligence (AI) technology in a single city often extends to surrounding regions through mechanisms such as knowledge diffusion and talent mobility. By leveraging these successful implementations, neighboring cities can accelerate their own technological advancements, collectively strengthening regional public health resilience (47).

## 6 Research conclusions

This study analyzes the impact of AI technology on urban public health resilience using a sample of 284 prefecture-level and above cities in China from 2011 to 2021. The findings reveal that: (1) AI technology significantly enhances urban public health resilience, primarily by strengthening the public health system’s resistance, recovery, and innovation capabilities. (2) A regional heterogeneity analysis shows that AI technology has a significant positive effect on public health resilience in the eastern and central regions. In particular, the eastern region benefits most from its strong technological absorption capacity and effective allocation of public health resources. However, in the western region, where economic development and technological foundations are weaker, AI technology has not demonstrated a significant effect in enhancing resilience. (3) The spatial spillover effects indicate that AI technology exhibits a significant positive spillover effect, especially in the indirect effects of patent authorizations. This suggests that authorized patents are more likely to be applied in neighboring cities, thereby improving the overall resilience of urban public health systems.

### 6.1 Theoretical and practical implications

This study highlights the pivotal role of artificial intelligence (AI) technology in bolstering urban public health resilience, offering valuable insights for policymakers seeking to enhance preparedness and response strategies. First, the accelerated development of AI technology is critical for mitigating external crises. AI-powered platforms for real-time health data collection and analysis can significantly improve the prediction and management of urban public health emergencies. Moreover, integrating AI into disease diagnosis, treatment planning, and the allocation of medical resources enhances healthcare efficiency and precision. Establishing dedicated funding streams and innovation hubs to support research and development in AI applications for public health is essential to sustain these advancements. Second, building human capital and ensuring a robust supply of AI talent are paramount. Tailored training programs for healthcare professionals and AI specialists should be implemented to enhance their technical expertise and interdisciplinary knowledge. Additionally, education initiatives that bridge urban public health, AI, and data science can foster the development of multifaceted professionals. Programs aimed at promoting diverse participation in AI research and applications are also necessary to ensure inclusivity and innovation in public health solutions. Third, robust intellectual property (IP) protection is indispensable. Simplifying and expediting patent application processes for AI and healthcare technologies can incentivize innovation. At the same time, strengthening enforcement mechanisms against IP violations offers crucial legal protections for innovators. Financial support and legal assistance should be extended to small and medium-sized enterprises to safeguard their AI-driven innovations in the public health sector. Finally, fostering cross-regional collaboration and resource sharing is a strategic imperative for enhancing public health resilience at a broader scale. Cities can work together through information exchange, technological partnerships, and the seamless flow of resources to strengthen collective preparedness and adaptability in neighboring urban areas.

### 6.2 Limitations and future directions

While this study confirms the positive impact of AI technology on urban public health resilience, several limitations remain. First, as the sample data primarily focus on 284 Chinese cities, the findings may be most applicable to the Chinese context. Future research should extend to developed countries in Europe and the United States, as well as other emerging economies, for comparative analysis. Second, this study uses patent applications and authorizations as the main indicators of AI technology. Future studies could incorporate additional dimensions (e.g., technology application levels, mobility of scientific talent) to provide a more comprehensive picture of the impact of AI technology on public health resilience. Finally, this study primarily explores the direct and spatial spillover effects of AI technology on public health resilience. Future research could further analyze the impact of factors such as transportation barriers, political boundaries, and cultural differences on urban public health resilience. It would also be valuable to consider using *effective distance* as a spatial weight matrix to avoid the potential inaccuracies associated with purely relying on geographic distance, which might lead to misleading results.

## Data Availability

The raw data supporting the conclusions of this article will be made available by the authors, without undue reservation.
